# Distinct genetic alterations in small cell carcinoma from different anatomic sites

**DOI:** 10.1186/2162-3619-4-2

**Published:** 2015-01-14

**Authors:** Xiaoyong Zheng, Delong Liu, John T Fallon, Minghao Zhong

**Affiliations:** Department of Pathology, Westchester Medical Center/New York Medical College, Valhalla, NY USA; Henan Tumor Hospital, Zhengzhou University, Zhengzhou, China

## Abstract

Small cell carcinoma (SmCC) is a distinct clinicopathological entity first described in the lung. It represents approximately 15% of all bronchogenic carcinoma. Extrapulmonary small cell carcinoma (EPSmCC) morphologically indistinguishable from small cell lung cancer (SCLC) was first reported in 1930. Since its first description, EPSmCC has been reported in virtually all anatomical sites, including: gynecologic organs (ovary and cervix); genitourinary organs (urinary bladder and prostate); the gastrointestinal tract (esophagus); skin (Merkel cell carcinoma) and head and neck region. Regardless of the anatomic sites, all SmCCs have similar, if not identical, histo-pathology features and immunohistochemical profile. SmCC is one of the most aggressive malignancies. The molecular mechanisms underlying its development and progression remain poorly understood. Herein, we reviewed the literature in SmCC in respect to its site of occurrence, clinical features, immunohistochemical characteristics. SmCCs have heterogeneous molecular mutations. Dinstinct genetic alterations associated with SmCC from different body sites were reviewed. Some genetic alterations such as *RB1*, *TP53* are commonly seen in different origins of SmCC. Other genes with site specificity were also summarized, such as bladder SmCC with *TERT* promoter mutations; prostate SmCC with *ERG* translocations; ovarian SmCC with *SMARCA4* mutations; Merkel cell carcinoma (skin) and cervical SmCC with Merkel cell polyomavirus (MCV or MCPyV) and human papillomavirus (HPV). Further studies are needed to employ a genetically oriented approach for the diagnosis and therapy of SmCC.

## Introduction

**Figure 1:**
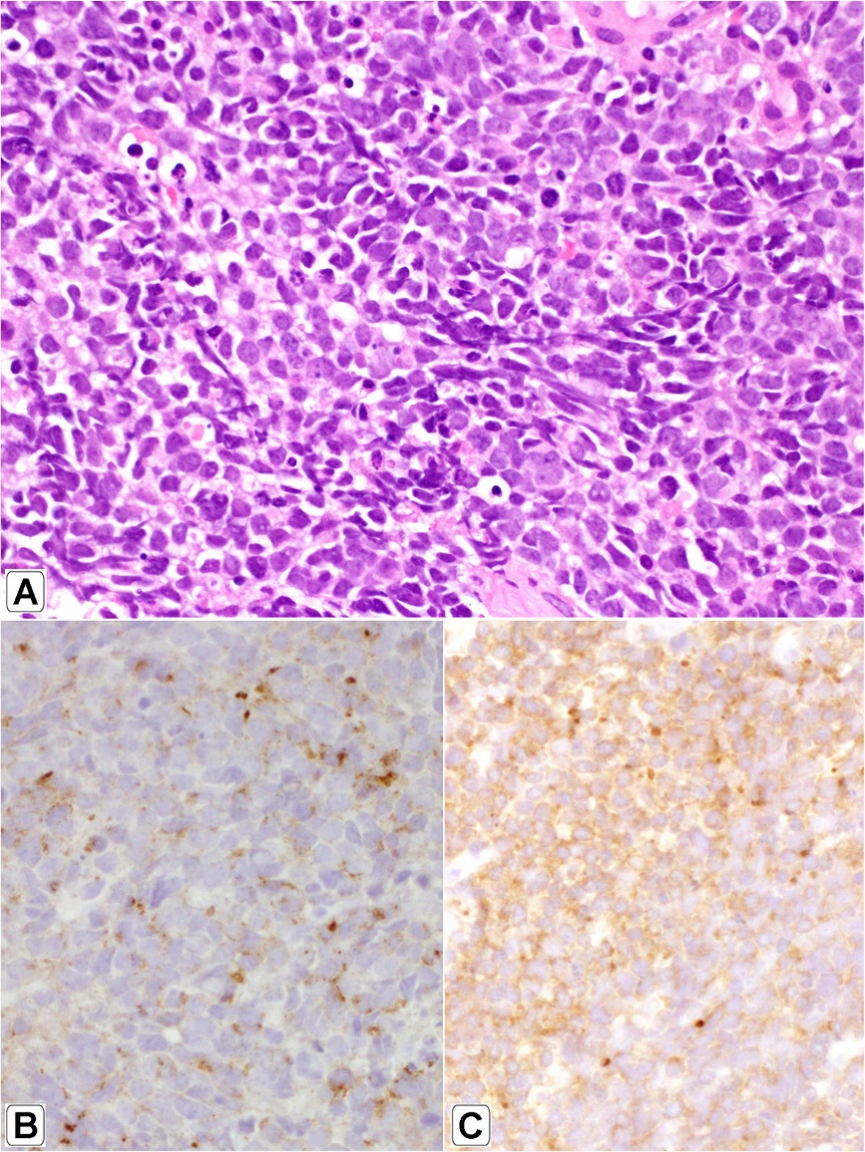
**Morphology of small cell carcinoma.** H&E staining x400 **(A)**; Immunohistochemistry of chromogranin **(B)** and synaptophysin **(C)**.

Small-cell carcinoma ("oat-cell carcinoma") is a type of highly malignant cancer that commonly arises in the lung. Uncommonly, small-cell carcinoma arising from outside of the lungs and pleura is referred to as extrapulmonary small-cell carcinoma (EPSmCC). The diagnosis of SmCC is primarily based on path-histologic criteria: sheets, ribbons, clusters, rosettes or peripheral palisading of small to medium sized (2-4x neutrophils) round/oval cells with minimal cytoplasm, salt and pepper chromatin without prominent clumps, hyperchromatic, indistinct nucleoli, nuclear molding, smudging, frequent mitotic figures (Figure 1A). In addition, SmCC cells show neuroendocrine differentiation and are positive for neuroendocrine tissue markers: chromogranin and synaptophysin (Figure 1B and C). The clinical behavior of SmCC from different anatomic sites are quite similar. SmCC cells usually metastasize very early, respond dramatically to chemotherapy (CT) and radiation therapy (RT) [1]. Patients usually have a very poor prognosis and short survival time despite treatment. Treatment of SmCC of lung and EPSmCC is similar. However, recent evidence at molecular and genetic levels suggests that SmCC from different anatomic sites may have distinct genetic biomarkers and is a heterogenous group of diseases. This conceptual change is critical for better understanding of these aggressive malignancies and may lead to a genetically oriented approach for the diagnosis and targeted therapy of SmCC.

### I. Small-cell lung cancer

Small-cell lung cancer (SCLC), representing 15% of all bronchogenic carcinoma cases, is a distinct subtype associated with a typical clinical picture of early metastasis. Chemotherapy alone or combined with radiation, but not surgery, is the usual treatment of choice for small cell lung cancer. On this regimen, a large percentage of patients experiences remission. The 5-year survival for small cell lung cancer (6%) is however much lower than that for non-small cell lung cancer (NSCLC) (18%). One major reason is that targeted therapy has been widely used for NSCLC treatment, and mutation analysis is routinely done now for EGFR, KRAS, or ALK. More and more novel agents for targeted therapy of NSCLC are being developed [2–4].

As for SmCC, several tumor suppressor genes are inactivated, including *TP53* (80–90% of cases, [5]) *RB1* (60–90% of cases [6, 7]) and *PTEN* (13% of cases [8]). In mice, SCLC is initiated by deletion of two tumor suppressor genes (*RB1* and *TP53*). Deletion of these two genes produces a model that recapitulates the clinical features of human SCLC. Infrequent activating mutations have also been found in *PIK3CA*, *EGFR* and *KRAS* (all 10% or lower). In addition, *MYC* is amplified in 20% of cases [9]. Mean levels of total PARP1 (a DNA repair protein and E2F1 co-activator) were higher in SCLC cell lines than in NSCLC cell lines, and SCLC growth was inhibited by PARP1 and EZH2 knockdown [10].

### II. Small cell carcinoma of genitourinary tract

The genitourinary tract is the most common extrapulmonary site for EPSmCC, with approximately 900 new cases diagnosed every year in the United States [11]. The most common sites for SmCC of the genitourinary tract are the urinary bladder and prostate; however, it is still very rare, accounting for only 0.7% and 0.5% of all bladder and prostate cancer, respectively. SmCC of the genitourinary tract is an aggressive cancer, with a poor prognosis overall. Although there is no standard of care, patients are treated using a multimodality approach analogous to those used in the treatment of small-cell lung cancer [12].

1. Renal SmCC is an extremely rare malignancy and accounts for less than 1% of all renal tumors. Although renal SmCC shares similar morphological and immunohistochemical features with SmCC of other organs, renal SmCC often (60-70%) coexists with conventional urothelial carcinoma, suggesting a potential association between SmCC and urothelial carcinoma in the kidney [13, 14]. Renal SmCC affects patients of various ages and appears to be more common in men (male–female ratio = 2:1) [14]. Its clinical presentations are similar to those of renal cell carcinoma or urothelial carcinoma, and it is histologically and immunohistochemically indistinguishable from its pulmonary or other EPSmCC counterparts. Most patients present at an advanced stage with widespread metastases and a dismal prognosis despite multimodal therapy. However, if the disease is found and treated early, long-term survival may be possible for patients with an organ-confined tumor [13].
2. Small cell carcinoma of the urinary bladder

SmCC of urinary bladder is a malignant neuroendocrine neoplasm derived from the urothelium which histologically mimics its pulmonary counterpart. A recent analysis from the Surveillance, Epidemiology, and End Results of the SmCC of the urinary bladder database indicated a significant rise in the incidence of the SmCC of the urinary bladder in the United States from 0.05 to 0.14 cases per 100,000 people between 1991 and 2005 [15]. This is likely due to increase in the U.S. population’s age. Similar to other bladder cancers, risk factors for SmCC of urinary bladder include smoking, male sex (male-female ratio of 3:1), and advanced age. The average age of incidence was found to be 71.7 ± 11.2 years, with a median age of 73 years [15]. Caucasians were most commonly affected, with a white-nonwhite ratio of 10:1. More than 60% of the SmCC of the lung has metastatic disease at the time of diagnosis. Similar rates have been shown in SmCC of the urinary bladder [11]. Chemotherapy is the mainstay of treatment, with proven survival benefit [16].

One study of immunohistochemical (IHC) stains in SmCC of urinary bladder has shown that nuclear GATA3 expression was encountered in 7 bladder (7/22, 32%), 2 lung (2/15, 13%), and 0 (0/33, 0%) prostate SmCC [17]. TTF-1 expression in SmCC of urinary bladder was found in 40% of the tumors in 2 studies, demonstrating that TTF1 can be expressed in EPSmCC [18, 19]. SmCC of urinary bladder are also stained positive with the epithelial markers: CAM 5.2, CK7, and EMA in 59%, 41%, and 77.7% of the cases, respectively. This supports the urothelial origin of SmCC of urinary bladder [18, 20]. Distingushing SmCC of the prostate and from that of the bladder can be very challenging, if even possible, because of low positivity of GATA3 in SmCC of the bladder and low positivity of PSA and other prostatic markers, such as P501S, in SmCC of the prostate [21, 22].

*TERT* promoter mutations, originally discovered in ~70% of melanomas, have also been found to be the most common form of genetic mutations in urothelial carcinomas. Interestingly, these mutations have very low incidence in other prevalent carcinomas: lung, prostate and colon cancers. Multiple studies [23–25], including our unpublished results, demonstrated that up to 70-80% of urothelial carcinoma carries the *TERT* promoter mutations irrespective of grade, stage or location. Our recent study [10] showed that 100% (10 cases) of SmCC of the urinary bladder carry *TERT* promoter mutation C228T, yet none of SmCC from all other origins including prostate, lung, cervix, esophagus, and skin (Merkel cell carcinoma) contain the *TERT* promoter mutations. This study indicated that the *TERT* promoter mutation may be a biomarker to distinguish SmCC of the urinary bladder from SmCC of other origins.

3. Small cell carcinoma of the prostate

SmCC of the prostate was first described by Wenk et al. [26] more than 30 years ago. Since then, it has been reported to occur in 0.5–2% of men with prostate cancer, although autopsy studies of men who have died of castration-resistant prostate cancer have reported the presence of SmCC in up to 10–20% of cases [27, 28]. Neuroendocrine markers such as chromogranin A and synaptophysin are expressed in nearly all cases of conventional prostatic adenocarcinoma, with the proportion of cells that stain positive for these markers increasing during castration [29]. In 24% and 35% of cases, p63 and high-molecular-weight cytokeratin were noted to be positive, which are typically negative in prostatic adenocarcinoma [21]. Studies have demonstrated thyroid transcription factor 1 (TTF-1) expression in over 50% of SmCC of the prostate, limiting its utility in distinguishing primary SmCC of the prostate from metastatic SmCC of the lung [22]. PSA and other prostatic markers, such as P501S, are only positive in about 17–25% cases, often focally [21, 22]. These results demonstrated that IHC stains have very limited value to discriminate SmCC of prostate from SCLC.

Interestingly, both conventional prostatic adenocarcinoma and SmCC of prostate share *ERG* gene rearrangement which is absent in SmCC from other body sites. This rearrangement occurs between an androgenregulated gene, *TMPRSS2* (21q22.3) and an *ETS* transcription factor family member, most commonly *ERG* (21q22.2), resulting in a gene fusion product, *TMPRSS2–ERG* gene fusions. This result not only indicates a common clonal origin between conventional prostatic adenocarcinoma and SmCC of prostate, but also implies the clinical use of *ERG* gene rearrangement as a biomarker to confirm a prostatic origin for SmCC [30, 31].

### III. Small cell carcinoma of gynecologic tract

The SmCC of gynecologic tract is one of the common EPSmCCs, representing up to 2% of all gynecologic malignancies [32, 33]. Reported gynecologic sites include the cervix, endometrium, ovary, fallopian tube, vagina and vulva.

1. Small cell carcinoma of the uterine cervix (SmCCC)

The uterine cervix is the most common gynecologic tract site involved with EPSmCCs. However, SmCCC is a very rare disease, representing only 1% to 3% of all uterine cervical cancers. SmCCC often coexists with conventional squamous cell carcinoma or adenocarcinoma. Depending on the series analyzed and the selection criteria employed, between 11% and 64% of SmCCC cases present admixed histology [34, 35]. Immunohistochemical studies have further revealed that the majority of cases show diffuse nuclear and cytoplasmic p16 positivity [36, 37].

The critical role of human papillomaviruses (HPV) in the carcinogenesis of conventional cervical cancer is well established. The prevalence of the different high-risk HPV types in SmCCC has been preliminarily established and reported to range from 50% to 100% [38, 39]. It has been found that unlike in squamous cell carcinoma of the cervix, HPV 18 may be the most prevalent type of SmCCC [37, 40]. Given this evidence, SmCCC, like other types of cervical cancer, seems to be associated with high-risk HPV infection. For the purpose of differential diagnosis, HPV is specific for SmCC of cervical origin other than ovary or lung, but p16 immunohistochemistry is not useful for this purpose [36].

2. Small-cell carcinoma of the ovary

Small-cell ovarian carcinoma is divided into two categories: pulmonary type (SCCOPT) and hypercalcemic type (SCCOHT). The cellular features and neuroendocrine markers of the pulmonary type resemble small-cell carcinoma of the lung, whereas the immunohistochemical markers and microscopic and ultrastructural examination of SCCOHT do not [41]. Both of these tumors are uncommon, but SCCOPT is extremely rare with only approximately 20 cases reported in the English literatures.

### SCCOPT

The mean age of diagnosis of patients with SCCOPT is 51 years (22-85 years) [42]. Bilateral disease is present in about half of the cases and all cases lack hypercalcemia [42]. TTF-1 was found to be diffusely positive in one case but absent in another case [36]. The diagnosis of CSCOPT is exclusion of metastasis of SmCC from other locations, in particular lung. One report showed that malignant transformation of ovarian mature cystic teratoma with a predominant pulmonary type small cell carcinoma component, is CDX2 positive [43].

### SCCOHT

SCCOHT represents less than 1% of all ovarian cancer diagnoses, with fewer than 300 cases reported in the literature thus far [44, 45]. The mean age of diagnosis is 23 years, and the majority of affected women present with early-stage disease. Nonetheless, most patients relapse and die within 2 years of diagnosis, regardless of tumor stage, with a long-term survival rate of only 33%, even when disease is confined to the ovary at diagnosis. The tumor appears nearly almost unilaterally, mostly affecting the right ovary [44, 46].

Recently 3 independent studies reported that SCCOHT is a monogenic disease caused by mutations in the *SMARCA4* gene. Whole-exome sequencing on DNA obtained from 24 familial or sporadic cases of SCCOHT revealed that 22 of the 24 cases analyzed were due to *SMARCA4* mutations; and Immunohistochemical analysis of these cases and additional familial and non-familial cases showed loss of SMARCA4 (BRG1) protein in 38 of 40 tumors [47]. Furthermore, the researchers suggest that SCCOHT tumors are essentially malignant rhabdoid tumors of the ovary, “they are not always comprised of small cells, are not carcinomas, and only two thirds have hypercalcemia”. It is, therefore, possible that chemotherapeutic regimens used to treat rhaboid tumors might help improve the outcome of this disease. Ramos et al. [48] reported that germline and somatic inactivating mutations in the SWI/SNF chromatin-remodeling gene *SMARCA4* in 75% (9/12) of SCCOHT cases in addition to SMARCA4 protein loss in 82% (14/17) of SCCOHT tumors but in only 0.4% (2/485) of other primary ovarian tumors. Witkowski et al also reported similar results. These new pieces of evidence demonstrated that alterations in *SMARCA4*, the major cause of SCCOHT, could lead to improvements in genetic counseling and new treatment approaches [46].

### IV. Merkel cell carcinoma (MCC)

MCC is a rare neuroendocrine tumor of the skin with rising incidence and an aggressive behavior. The annual incidence of MCC is 0.6 per 100,000 persons and is increasing (approximately 1,600 new cases per year in the US) [49]. Histologically, MCC shares numerous features with SmCC. Immunohistochemically, MCC stains positive for synaptophysin and chromogranin [50]. cytokeratin CK-20 is positive in 89–100% of Merkel cell tumors with a punctate pattern and may be used to distinguish MCC from other tumor types. However, 33% of small cell lung cancers (SCLCs) and 3–4% of EPSmCCs also stain positively for CK20 [51].

Merkel cell polyomavirus (MCV), a new human polyomavirus, is clonally integrated in 70–80% of Merkel cell carcinoma (MCC) tumors. MCV is part of the normal, healthy skin flora but causes cancer after viral genome mutations eliminate its replication capacity. While similar to known polyomaviruses, MCV oncogenes act in new ways, such as activation of the survivin oncoprotein and PP2A-independent targeting of cap-dependent translation [52, 53]. Survivin inhibition improves survival of mice bearing human MCC xenografts [54].

### V. The origin of small cell carcinoma

The identification of the cell type(s) from which small cell carcinoma originates is critical in the development of methods for early diagnosis and treatment.

1. The cell of origin for small cell lung cancer

The usage of cell type-restricted Adeno-Cre vectors to distinct cell populations in the lung of adult mouse showed that loss of *TP53* and *RB1* can efficiently transform neuroendocrine and Surfactant Protein C (SPC)-expressing cells to SCLC, albeit SPC-expressing cells at a lesser efficiency. In contrast, Clara cells were largely resistant to transformation. These results indicate that although neuroendocrine cells serve as the predominant cell of origin for SCLC, a subset of SPC-expressing cells may also be endowed with this ability [55, 56].

2. The cell of origin for EPSmCCs

The origin of EPSmCCs is controversial. It was assumed that these neoplasms arise from neuroendocrine cells in the Amine Precursor Uptake and Decarboxylation (APUD) system [57, 58]. To date, however, it is thought that the origin of EPSmCCs is either a totipotent stem cell capable of differentiating into a variety of cell types, or that elements of SmCC arise as a late-stage phenomenon in the genetic progression of carcinomas [57]. The presence of mixed carcinomas may have implications for the origin of EPSmCC, which may arise from multipotent stem cells that retain the ability to differentiate into various tissue types. Evidence for this hypothesis comes from an identical pattern of allelic loss in SmCC of urinary bladder mixed with urothelial carcinoma (UC) [59]. For bladder localizations, a malignant transformation of the neuroendocrine cells physiologically located in the urothelium has been proposed [60]. Molecular genetic studies have also suggested a common clonal origin for the coexisting bladder SmCC and conventional UC [59, 61]. X chromosomal inactivation analysis [59] in females illustrated the same nonrandom inactivation in both SmCC of the urinary bladder and UC. Identical point mutations of *TP53* were found in invasive bladder SmCC and coexisting UC in situ; additionally, no loss of heterozygosity of 9 microsatellite markers and *TP53* was found in either component. This study provided evidence for the development of bladder SmCC out of bladder UC in situ [61].

Other theories of histogenesis include metaplasia from other high-grade malignancies. Neuroendocrine (NE) cells are detected by IHC in some in-situ cervical adenocarcinomas, and these may be the origin of cervical NE carcinomas [62]. Further support for this theory comes from a study where identical loci with loss of heterozygosity were demonstrated in mixed adenocarcinoma and EPSmCC of the appendix, and an additional locus was found in the EPSmCC component [63]. Similarly, additional loci with loss of heterozygosity were noted in breast EPSmCC compared with adjacent ductal carcinoma in situ.

There is a high concordance rate of *ERG* rearrangement between the SmCC of prostate and prostatic acinar components in a given patient; however, the absence of *ERG* rearrangement in bladder or lung small cell carcinomas supports a common origin for these two subtypes of prostate cancer.

In one report, malignant transformation of ovarian mature cystic teratoma consists of a predominant pulmonary type SmCC component (65%), as well as minor components including adenocarcinoma (25%), squamous cell carcinoma (5%), and transitional cell carcinoma (5%). CDX2 positivity was retained in all of the carcinomas. It may suggest that the adenocarcinoma had arisen from an intestinal epithelium in the mature cystic teratoma and then differentiated into the diverse histological types mentioned above [43].

Recent reports [46, 48] also revealed that small cell carcinoma of the ovary, hypercalcemic type, display frequent inactivating germline and somatic mutations in *SMARCA4*, a mutation rare in other common tumors. It has been well established that Merkel cell virus is strongly associated with only Merkel cell carcinoma, but not SmCC of any other origins [52].

Loss of RB1 function is associated with the development of neuroendocrine tumors, including prostate, pituitary, thyroid and adrenal gland tumors [64–67]. This mutation may give this type of cancer mutual morphologic features. Loss of RB1 by deletion is a common event in prostatic small cell carcinoma (90, 26/29), but rarely occurs in high-grade acinar tumors (7%, 10/150) and primary acinar carcinomas with neuroendocrine differentiation (11%, 4/35) [68]. General inactivation of the RB1 pathway and deregulation of the cell cycle was a common early event in human cancers [69, 70], however homozygous deletion of RB1 is a relatively late-stage genomic alteration in acinar prostate cancer progression [68].

These results suggested that at least some EPSmCC has the same cell origin of conventional carcinoma, such as urothelial carcinoma, prostatic adenocarcinoma, et al.

## Conclusion

Current evidences strongly suggest that despite significant overlap of morphology and immunophenotype among SmCCs from different anatomic sites, SmCCs have heterogeneous molecular mutations (Table 1). A genetically oriented approach for the diagnosis and therapy of SmCCs becomes necessary for significant clinical impact on the prognosis and therapy outcome of this heterogeneous group of aggressive malignancies.

**Table 1:** **Gene mutations in small cell carcinoma**

Location	Genes	Small cell carcinoma	Non-small cell carcinoma	Notes
Lung	*TP53 mutation*	80–90% [5]	40-60%	
	*RB1 mutation*	60–90% [71]	15-30%	
	*PTEN mutation*	60-90% of cases [8]	40%	
	PARP1 high expression	2.6 fold higher than non-Small cell carcinoma [10]		PARP-1 inhibitors as anti-cancers
Urinary bladder	*TERT* promoter mutation	100%	60-70%	Not see in SmCC from prostate, lung, ovary, or esophagus [10]
	*TP53*	overexpression 54% p53 negative staining 46% [72]	*TP53* mutations 14% 30%	
Prostate	*ERG* gene rearrangements	45% [30, 31]	40-60% [63]	True prostate cancer specific biomarkers: PCA3 and TMPRSS2:ERG gene fusion [73]
	(*TMPRSS2–ERG* gene fusions)			
	*RB1* loss	90% [68]	34% of primary 74% of met [14]	Loss of *RB*1 function late in prostate cancer, early in other common cancers
	*RAS/RAF*	No report	43% of primary, 90% of met [14]	
	*PTEN*	63%[68]	4% of primary, 58% of met [14]	
SCCOHT	*SMARCA4* mutations	75-100% [46–48]	Very rare in other tumor [46–48]	Characteristic mutation in SCCOHT
Merkel cell carcinoma	MCV clonally integrated	80–97% [52–54]	8-16% of other tumor [52–54]	Characteristic integration in MCC
